# Right Pleuro-Pericardial Window during Cardiac Surgery: A Safe and Simple Technique that Decreases Postoperative Atrial Fibrillation

**DOI:** 10.5761/atcs.oa.25-00102

**Published:** 2025-08-16

**Authors:** Nadine Kawkabani, Rita Farah, Joseph Akar, Wael Daajeh, Mohammad Mokdad, Moussa Abi Ghanem, Bassam Abi Khalil

**Affiliations:** 1 Saint George Hospital-University Medical Center, Beirut, Lebanon; 2 Inspect-LB: Institut National de Sante Publique, Epidemiologie Clinique et Toxicologie, Beirut, Lebanon; 3 Division of Medical Toxicology, Department of Emergency Medicine, University of Virginia School of Medicine, Charlottesville, VA, USA; 4 Section of Cardiovascular Medicine, Yale School of Medicine, New Haven, CT, USA

**Keywords:** cardiac surgery, atrial fibrillation, pleuropericardial window

## Abstract

**Purpose:** Postoperative atrial fibrillation (POAF) is a common complication following cardiac surgery and is associated with increased hospital stay, morbidity, and mortality. One of the major factors predisposing patients to the development of POAF is inflammation related to pericardial effusions, which may occur after cardiac surgery. We hypothesized that by creating a pleuro-pericardial window before closing the chest during cardiac surgery, draining the pericardial space into the right pleural space may lead to fewer pericardial effusions and less postoperative atrial fibrillation.

**Methods:** We conducted a study that included 172 consecutive patients (67 ± 12 years, 48.3% female) who underwent cardiac surgery (73.8% aortic valve replacement [AVR], 5.8% mitral valve replacement, 19% AVR + coronary artery bypass grafting). The first 95 patients included in this study (67 ± 12 years, 48% female) did not have any pleuro-pericardial window created, whereas the remaining 77 patients (67 ± 12 years, 48% female) did. Baseline clinical and procedural characteristics were compared between the 2 groups. Postoperative events and complications were collected until hospital discharge.

**Results:** A total of 50 patients developed POAF (29%). The incidence of POAF among patients who underwent a pleuro-pericardial window was 18.2% (95% confidence interval [CI]: 9.4%–27.0%). The incidence of POAF among those who underwent the standard technique was 37.7% (95% CI: 28.0%–47.8%). The patients who underwent a pleuro-pericardial window had a higher incidence of dyslipidemia (p = 0.037), right bundle branch block (p = 0.018), 1st-degree atrioventricular block (p = 0.046), and previous myocardial infarction (p = 0.006). Multivariate analysis showed that the risk of POAF was significantly lower in patients who underwent a pleuro-pericardial window compared to those who did not (odds ratio: 0.46, 95% CI: 0.24–0.87, p = 0.019).

**Conclusion:** Creating a right pleuro-pericardial window before closing the chest after cardiac surgery was independently associated with a lower incidence of POAF.

## Introduction

Atrial fibrillation is a common complication after cardiac surgery and is associated with increased hospital stay, morbidity, and mortality.^1)^

The pathophysiology of postoperative atrial fibrillation (POAF) is multifactorial. Electrolyte imbalance, transient hypoxemia, electrophysiological disturbances, atrial stretching, and inflammation related to pericardial effusions may lead to POAF.^1)^

We hypothesized that draining the pericardial space into the right pleura before closing the chest would decrease the incidence of POAF in cardiac surgery patients.

## Materials and Methods

### Study design and population

We conducted a retrospective study that included 172 consecutive patients who underwent cardiac surgery by a single surgeon at our hospital between January 1, 2016 and March 31, 2023. Patients with a history of atrial fibrillation and patients requiring left pleura opening during surgery were excluded. Patients were divided into 2 groups. In one group, a right pleuro-pericardial window was created before closing the chest, and in the 2nd group, no window was done (the right pleura was not opened). The incidence of POAF was measured. The study was approved by the Institutional Review Board.

To note, the right pericardial window was achieved by opening the right pleura starting at the mid-sternal level and proceeding caudally toward the diaphragm. The opening was continued perpendicularly at the diaphragmatic level through the pericardium toward the inferior vena cava. A fat pad is usually present in this area and can be resected when needed. This surgical procedure creates a window that drains the whole pericardial space into the right pleura.

Baseline characteristics including clinical (demographics, history, and risk factors) and procedural factors were described. Postoperative events and complications were collected until hospital discharge.

### Statistical analysis

We analyzed data using SPSS 23.0 (BM SPSS Statistics for Windows, Version 23.0; IBM Corp., Armonk, NY, USA). Means with standard deviations and frequencies with percentages were used to describe continuous and categorical variables, respectively. We used the Pearson chi-square test to compare categorical variables and Student’s t-test to compare means between 2 groups. We studied the association of POAF with the type of procedure (performing a right pleuro-pericardial window or not) using a binary logistic regression. Clinical characteristics that showed statistical differences in the bivariate analysis were entered into the multivariate analysis. The level of significance was set at p <0.05.

## Results

A total of 172 patients were included in this study (67 ± 12 years, 48.3% female). Patients underwent aortic valve replacement (AVR) (n= 127, 73.8%), combined surgery (coronary artery bypass grafting [CABG] + AVR) (n = 33, 19%), and mitral valve replacement (n = 10, 5.8%).

Among these patients, the first 95 patients included in this study (67 ± 12 years, 48% female) did not have any pleuro-pericardial window created before closing the chest, whereas the remaining 77 patients (67 ± 12 years, 48% female) did. A total of 50 patients developed POAF (29%).

The incidence of POAF among patients who underwent the standard technique was 37.7% (95% confidence interval [CI]: 28.0%–47.8%). Baseline clinical and procedural characteristic comparison between the 2 groups is summarized in **Table 1**.

**Table 1:** Demographic, clinical, and laboratory characteristics by type of procedure: bivariate analysis

	Surgery with PPW creation (N = 77)	Surgery without PPW creation (N = 95)	p
Age (years), mean (standard deviation)	66.8 (11.5)	67.2 (11.9)	0.4
Gender			0.96
Male	40 (52.0)	49 (51.6)	
Female	37 (48.1)	46 (48.4)	
Diabetes mellitus	23 (29.9)	28 (29.4)	0.58
Hypertension	62 (80.5)	69 (72.6)	0.17
Dyslipidemia	46 (59.7)	43 (45.3)	0.037
Previous myocardial infarction	8 (10.4)	1 (1.1)	0.006
Chronic renal failure	1 (1.3)	4 (4.2)	0.38
Congestive heart failure	6 (7.8)	2 (2.1)	0.14
Pulmonary hypertension	5 (6.5)	5 (5.3)	0.74
Beta blockers	46 (59.7)	55 (57.9)	0.8
Calcium channel blockers	10 (13.0)	26 (27.4)	0.024
ACE inhibitors	23 (29.9)	18 (18.9)	0.09
Atrioventricular block	9 (11.7)	4 (4.2)	0.046
Right bundle branch block	9 (11.7)	3 (3.2)	0.018
Left bundle branch block	5 (6.5)	2 (2.1)	0.13
Left anterior hemiblock	1 (1.3)	0	0.24
Ejection fraction <50%	9 (11.7)	4 (4.2)	0.088
Left atrium dilated	13 (29.9)	10 (10.5)	0.46
Annular dilation	4 (5.2)	2 (2.1)	0.4
Annular calcification	44 (57.1)	55 (57.9)	0.97
Bicuspid	6 (7.8)	17 (17.9)	0.042
Low potassium	4 (5.2)	4 (4.2)	0.7
Low magnesium	1 (1.3)	1 (1.1)	1
pH <7.36	10 (13.0)	8 (8.4)	0.4
Surgery			0.4
AVR	54 (70.0)	73 (76.8)	
AVR + CABG	16 (20.8)	17 (17.9)	
MVR	5 (6.5)	5 (5.3)	
Clamp time, median (interquartile range)	54 (48.7–65)	54 (50–67)	0.9
Pump time, median (interquartile range)	73 (67–88)	70 (65–84)	0.3

AVR: aortic valve replacement; CABG: coronary artery bypass grafting; MVR: mitral valve replacement; PPW: pleuro-pericardial window; ACE: angiotensin-converting enzyme

The patients who underwent a pleuro-pericardial window had a higher incidence of dyslipidemia (p = 0.037), right bundle branch block (p = 0.018), 1st-degree atrioventricular block (p = 0.046), and previous myocardial infarction (p = 0.006).

Multivariate analysis showed that the risk of POAF was significantly lower in patients who underwent a pleuro-pericardial window compared to those who did not (odds ratio [OR]: 0.46; 95% CI: 0.24–0.87; p = 0.019) (**Tables 2** and **3**).

**Table 2:** Incidence of POAF by surgical procedure

	Surgery with PPW creation	Surgery without PPW creation
Aortic valve replacement	18.5% (95% CI: 7.8%–29.2%)	39.7% (95% CI: 28.2%–51.2%)
Coronary artery grafting	25.0% (95% CI: 1.1%–48.8%)	17.6% (95% CI: 2.6%–37.9%)
Mitral valve replacement	0%	80.0% (95% CI: 24.5%–135.0%)

PPW: pleuro-pericardial window; CI: confidence interval; POAF: postoperative atrial fibrillation

**Table 3:** Association between postoperative atrial fibrillation and “procedure” using a binary logistic regression

	Odds ratio	95% Confidence interval	p
Technique (new vs. standard)	0.461	0.24–0.87	0.019
Previous myocardial infarction	1.7	0.2–12.6	0.6
Calcium channel blockers	1.9	0.2–1.2	0.1

## Discussion

In this study, the incidence of POAF was significantly lower in the group of patients who had a pleuro-pericardial window created before closing the chest.

Atrial fibrillation occurs in 30%–40% of patients after cardiac surgery and is associated with complications and adverse events.^2)^

Mechanical compression or local inflammation in close proximity to the atria, generated by even a small pericardial collection or thrombi, may precipitate atrial fibrillation after cardiac procedures.^3)^

Thus, draining the pericardial space after surgery may decrease effusions and lessen the occurrence of POAF.

Left posterior pericardiotomy was first described by Mulay et al. in 1995. They reported a significant reduction in the incidence of pericardial effusions and supraventricular arrhythmias postoperatively in 100 patients who underwent bypass surgery.^4)^

Gaudino et al found that creation of a posterior left pericardiotomy helped drain fluids and thrombi from the pericardial cavity into the left pleural space and was associated with a significant decrease in the incidence of POAF in patients undergoing CABG, AVR, and aortic operations without increasing complications related to this technique.^5)^

Hu et al. published a meta-analysis of 10 small randomized trials that compared left posterior pericardiotomy with no intervention and reported a significant reduction in the risk of POAF in the group of patients who underwent posterior left pericardiotomy. However, there was a considerable heterogeneity in the inclusion criteria, outcomes, definitions, and assessments.^6)^

Posterior pericardiotomy is currently recommended in the American College of Cardiology (ACC) guidelines since it appears to have higher efficacy, fewer side effects, and lower costs than other available options (prophylactic uses of amiodarone, beta blockers, magnesium, steroids, overdrive atrial pacing, etc.).^7)^

However, this technique necessitates tilting the heart to the right side of the chest, which may compromise some posterior structures (phrenic nerve, descending aorta, etc.) and is not recommended after valve procedures. This technique also cannot be performed in minimally invasive surgeries (partial sternotomies, right thoracotomies, etc.).

To note, one case of graft herniation through the pericardiotomy has been previously reported, resulting in postoperative myocardial ischemia. Excessive length of the vein graft has been incriminated.^8)^

Opening the right pleura and creating a large right pleuro-pericardial window, as shown in this paper, resulted in a significant decrease in POAF (18%). This procedure is easy to perform in all patients, especially in those undergoing minimally invasive procedures (partial sternotomies, right thoracotomies) and mitral valve surgeries. It does not necessitate any tilting of the heart, is achieved after coming off bypass, and does not require any additional resources. No complications related to this intervention nor pulmonary adverse events such as major atelectasis, pneumonia, significant pleural effusion, or acute respiratory distress syndrome were reported.

This technique should be the preferred procedure, specifically in cases where the left pleura is not opened. To note, we are presently studying the impact of creating a right pleuro-pericardial window on POAF in patients who also require opening the left pleura.

### Limitations

This study has some limitations. First, it was conducted in a single center, and the sample size is relatively small, resulting in a limited power for subgroup analysis. Second, due to the retrospective nature of the study, we can only suggest associations and cannot prove causality.

### Strengths

However, our results must be viewed and taken into consideration since the study was conducted in one of the major tertiary care hospitals in Lebanon. Furthermore, our trial excluded patients with a previous history of atrial fibrillation and patients who underwent procedures with left pleura opening.

## Conclusion

Creating a right pleuro-pericardial window before closing the chest in cardiac surgery procedures significantly decreases the incidence of POAF. It is a safe, inexpensive, and reproducible technique. However, larger and multicenter studies are needed to confirm its clinical benefits in cardiac surgery.

## Declarations

### Funding

None declared.

### Author contributions

All authors and co-authors (NK, RF, JA, WD, MM, MAG, and BAK) have contributed actively to this work. They have also read and approved the final version of this manuscript.

### Data availability

The data of this paper are available from the corresponding author upon reasonable request.

### Ethics approval and consent to participate

This study was approved by the Institutional Review Board at Saint George Hospital, University Medical Center.

### Consent for publication

Not applicable.

### Disclosure statement

The authors have nothing to declare.

## References

[1] Gaudino M Di Franco A Rong LQ Postoperative atrial fibrillation: from mechanisms to treatment. Eur Heart J 2023; 44: 1020–39. 36721960 10.1093/eurheartj/ehad019 PMC10226752

[2] Dobrev D Aguilar M Heijman J Postoperative atrial fibrillation: mechanisms, manifestations and management. Nat Rev Cardiol 2019; 16: 417–36. 30792496 10.1038/s41569-019-0166-5

[3] St-Onge S Perrault LP Demers P Pericardial blood as a trigger for postoperative atrial fibrillation after cardiac surgery. Ann Thorac Surg 2018; 105: 321–8. 29174782 10.1016/j.athoracsur.2017.07.045

[4] Mulay A Kirk AJ Angelini GD Posterior pericardiotomy reduces the incidence of supra-ventricular arrhythmias following coronary artery bypass surgery. Eur J Cardiothorac Surg 1995; 9: 150–2. 7786532 10.1016/s1010-7940(05)80063-6

[5] Gaudino M Sanna T Ballman KV Posterior left pericardiotomy for the prevention of atrial fibrillation after cardiac surgery: an adaptive, single-centre, single-blind, randomised, controlled trial. Lancet 2021; 398: 2075–83. 34788640 10.1016/S0140-6736(21)02490-9

[6] Hu XL Chen Y Zhou ZD Posterior pericardiotomy for the prevention of atrial fibrillation after coronary artery bypass grafting: a meta-analysis of randomized controlled trials. Int J Cardiol 2016; 215: 252–6. 27128541 10.1016/j.ijcard.2016.04.081

[7] Joglar JA Chung MK Armbruster AL 2023ACC/AHA/ACCP/HRS Guideline for the diagnosis and management of atrial fibrillation: A Report of the American College of Cardiology/American Heart Association Joint Committee on Clinical Practice Guidelines. Circulation 2024; 149: e1–156. 38033089 10.1161/CIR.0000000000001193 PMC11095842

[8] Yorgancioğlu C Farsak B Tokmakoglu H An unusual experience with posterior pericardiotomy. Eur J Cardiothorac Surg 2000; 18: 727–8. 11221729 10.1016/s1010-7940(00)00586-8

